# Increasing situational awareness through nowcasting of the reproduction number

**DOI:** 10.3389/fpubh.2024.1430920

**Published:** 2024-08-21

**Authors:** Andrea Bizzotto, Giorgio Guzzetta, Valentina Marziano, Martina Del Manso, Alberto Mateo Urdiales, Daniele Petrone, Andrea Cannone, Chiara Sacco, Piero Poletti, Mattia Manica, Agnese Zardini, Filippo Trentini, Massimo Fabiani, Antonino Bella, Flavia Riccardo, Patrizio Pezzotti, Marco Ajelli, Stefano Merler

**Affiliations:** ^1^Center for Health Emergencies, Bruno Kessler Foundation, Trento, Italy; ^2^Department of Mathematics, University of Trento, Trento, Italy; ^3^Department of Infectious Diseases, Istituto Superiore di Sanità, Rome, Italy; ^4^Covid Crisis Lab, Bocconi University, Milan, Italy; ^5^Department of Social and Political Sciences, Bocconi University, Milan, Italy; ^6^Laboratory for Computational Epidemiology and Public Health, Department of Epidemiology and Biostatistics, Indiana University School of Public Health, Bloomington, IN, United States

**Keywords:** reproduction number, situational awareness, epidemic surveillance, nowcasting, outbreaks

## Abstract

**Background:**

The time-varying reproduction number R is a critical variable for situational awareness during infectious disease outbreaks; however, delays between infection and reporting of cases hinder its accurate estimation in real-time. A number of nowcasting methods, leveraging available information on data consolidation delays, have been proposed to mitigate this problem.

**Methods:**

In this work, we retrospectively validate the use of a nowcasting algorithm during 18 months of the COVID-19 pandemic in Italy by quantitatively assessing its performance against standard methods for the estimation of R.

**Results:**

Nowcasting significantly reduced the median lag in the estimation of R from 13 to 8 days, while concurrently enhancing accuracy. Furthermore, it allowed the detection of periods of epidemic growth with a lead of between 6 and 23 days.

**Conclusions:**

Nowcasting augments epidemic awareness, empowering better informed public health responses.

## Background

Epidemiological surveillance is a critical tool for policy making, allowing public health professionals to monitor epidemic trends and the effectiveness of the adopted interventions. One important quantity that can be monitored during an epidemic outbreak by relying on surveillance system data is the time-varying reproduction number (*R*) (1). The reproduction number is defined as the average number of secondary infections caused by an average infectious individual and represents a summary metric that measures changes in transmissibility over time, indicating whether and how fast an epidemic is growing (when *R*>1) or declining (when *R* < 1). *R* can be estimated with established statistical methods (2–5) from the time series of the number of cases occurring in a given geographic unit (also known as “epidemic curve”), provided that the generation time distribution of the considered infection is known.

Surveillance systems are in most cases unable to trace the date at which cases were infected, and the temporally closest proxy event that can be measured is the onset of symptoms. Therefore, estimating *R* from epidemic curves organized as number of cases by date of symptom onset provides the closest estimate in time to the actual transmission events, even when symptomatic cases represent a small subset of all identified cases (6). Indeed, it has been shown that estimates of the reproduction number are robust as long as the proportion between symptomatic cases and total infections remains stable or even drifts slowly over time (5, 7).

Between the onset of symptoms for a patient and the insertion of their record in the surveillance database, temporal delays occur depending on the medical-seeking behavior of the individual, the logistics of case ascertainment (e.g., testing), the organization of healthcare response systems (e.g., administration of epidemiological questionnaires, contact tracing), and the socio-technological infrastructure for data collection, quality control, data upload and integration. These delays may change over time not only as a function of the progressive improvement of organizational aspects as the outbreak develops but also depending on the saturation of resources for diagnosis, contact tracing, data collection and transmission, and on other factors such as the population's perceived importance of medical seeking at different stages of the epidemic. As much as surveillance systems can be optimized to minimize some components of these delays, their data provide information that is always somewhat lagged with respect to the current epidemiological situation. The above-mentioned delays result in an underestimation (right-truncation) of epidemic curves for symptom onset dates close to the date of reporting, and data relative to these dates will consolidate only in successive updates of the surveillance system. This eventually limits the ability of public health officers to promptly assess the current situation or the effectiveness of recently implemented interventions.

If information on data consolidation delays is available, it can be exploited to “nowcast” epidemic curves, i.e. to adjust for right-truncation. The first methods were proposed in the wake of the AIDS pandemic (8–12), and further approaches were proposed in later years (13–22). The COVID-19 pandemic provided further momentum to this research topic (23–26) which also found application during the 2022 mpox epidemic (27, 28). Even though nowcasting may partially compensate the lack of knowledge on recent “occurred but not reported events” (13), in practical terms the right-truncation of incidence curves makes the following two questions of critical importance for real-time monitoring purposes: (i) until what date in the past can the epidemic curve (and therefore *R* estimates) be trusted, and (ii) how the incompleteness in recent data affects the accuracy of *R* estimates. In this study, we retrospectively assess the application of a simple nowcasting algorithm from a point of view of the improvement in situational awareness during an actual health emergency, using extensive surveillance data from the COVID-19 epidemic in Italy collected over more than 18 months.

## Methods

### Nowcasting algorithm

The nowcasting algorithm implemented here is a variant of the simple non-parametric method proposed by Lawless (13) and was independently developed for surveillance purposes during the early stages of the COVID-19 pandemic. If *C*_*D*_(*t*) is the epidemic curve as reported in the surveillance database at a given reporting date *D*, and C^*^(*t*) is the consolidated epidemic curve that will be reported at the end of the outbreak, the following relationship holds:


$$\begin{array}{l} C_{D}\left(t=D-z\right)=\pi_{D}\left(z\right){\cdot C}^{*}\left(t=D-z\right) \end{array}$$

where π_*D*_(*z*) is a “consolidation distribution” representing the degree of completeness in reported cases within *z* days from symptom onset estimated at time *D*. Equivalently, π_*D*_(*z*) can be interpreted as the cumulated probability distribution of a symptomatic case to be reported *z* days after symptom onset at time *D*. From this relationship, an estimate of the consolidated epidemic curve can be obtained from the reported epidemic curve if π_*D*_(*z*) is known. An approximation of π_*D*_(*z*) can be obtained by comparing successive updates of epidemic curves (13) over a window of recent epidemic curves with respect to *D*; in particular, we propose to approximate π_*D*_(*z*) with the average over observed consolidation distributions relative to *N* symptom onset dates closest to *D* and that can be considered consolidated at *D* (see Supplementary material for a formal and full description of the method). From π_*D*_(*z*) we can also define a “consolidation lag” *T*_*D, F*_ as the minimum number of days that will elapse before the completeness in reported cases exceeds a given fraction *F* of the final count. Note that π_*D*_(*z*) and *T*_*D, F*_ are continuously re-updated at every reporting date *D*, thereby implicitly keeping track of possible temporal changes in consolidation delays due to changes in the surveillance system or in the epidemiology of the pathogen under scrutiny.

We applied the algorithm to data on confirmed symptomatic SARS-CoV-2 infections collected by regional health authorities in Italy and collated by the Istituto Superiore di Sanità (Italian National Institute of Health) within the Italian COVID-19 integrated surveillance system (29) (a description of the system is reported in the Supplementary material). Here, we used the national-level epidemic curves by date of symptom onset as reported daily between May 1, 2020, and December 31, 2021. Epidemic curves between May 1, 2020 and June 28, 2020 were used to obtain the first stable estimates of the consolidation distribution; therefore, the algorithm was applied to *n* = 551 epidemic curves reported dates between June 29, 2020 and December 31, 2021.

For each of the *n* reporting dates *D*, we estimated the mean values of the time-varying reproduction number computed from non-adjusted epidemic curves [*R*_*D*_(*t*), or “net reproduction number”], and from nowcasted ones [${\hat{R}}_{D}\left(t\right)$, or “nowcasted reproduction number”], evaluated at dates *D*−*T*_*D, F*_ (with *F* ranging between 10 and 90% at intervals of 10%). The two estimates were then compared with corresponding estimates obtained from a consolidated epidemic curve reported several months after the end of the study period [R^*^(*t*), or “reference reproduction number”], evaluated at the same date. For each level of completeness *F*, we denote with **R**_*F*_, ${\hat{\text{R}}}_{F}$ and ${\text{R}}_{F}^{*}$, respectively the vectors of estimates *R*_*D*_(*D*−*T*_*D, F*_), ${\hat{R}}_{D}\left(D-T_{D,F}\right)$ and $R_{D}^{*}\left(D-T_{D,F}\right)$, obtained for different values of *D*.

### A practical example

**Figure 1:**
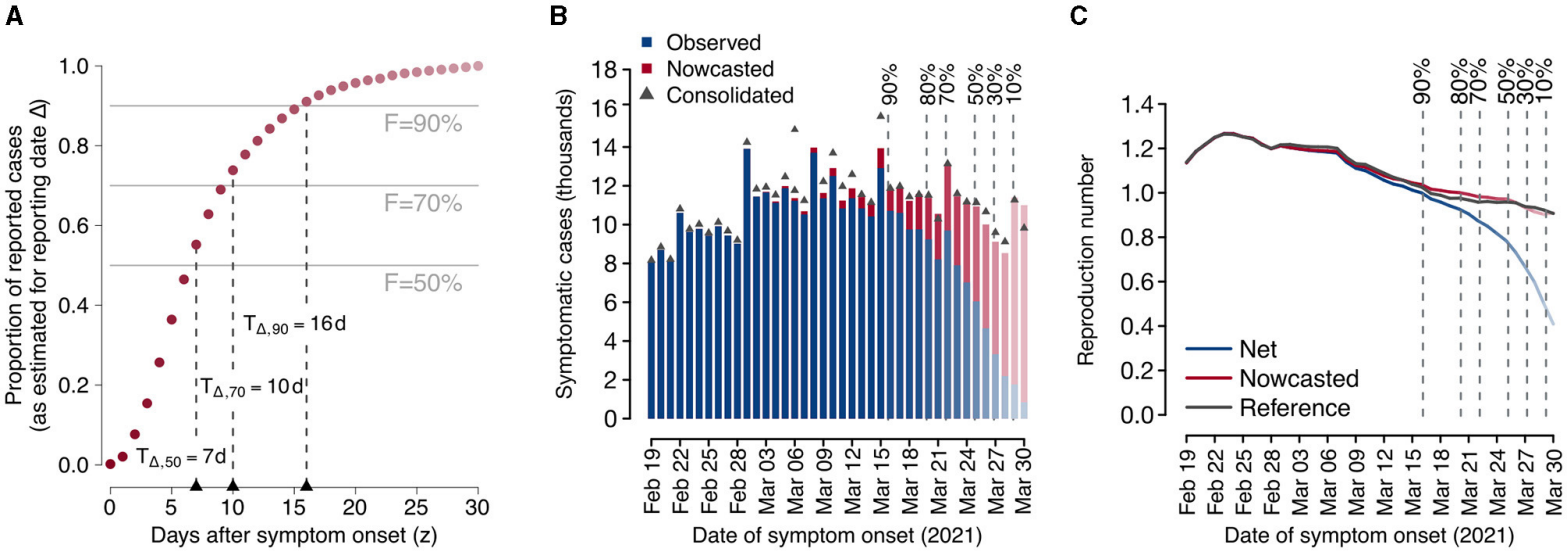
Example of application of the proposed nowcasting technique using data from the Italian COVID-19 integrated surveillance system for a selected reporting date (Δ = April 1, 2021). **(A)** Estimated consolidation distribution and corresponding consolidation lags. Dark red dots represent the estimated consolidation distribution at Δ. Horizontal lines define selected completeness thresholds (50, 70, and 90%) and vertical lines define the corresponding consolidation lags. **(B)** Observed and nowcasted epidemic curves by date of symptom onset. The consolidated epidemic curve is shown as dark gray triangles. Vertical dashed lines show the dates at which the observed number of cases is estimated to have reached a given completeness value. Bars in the epidemic curve are reported in fading colors with a level of darkness proportional to the estimated completeness. **(C)** Mean estimates of the net, nowcasted, and reference reproduction numbers over time. Vertical dashed lines show the dates at which the observed number of cases is estimated to have reached a given completeness value. The level of darkness in line colors is proportional to the estimated completeness.

Figure 1 reports a practical example of the proposed method for a specific reporting date Δ= April 1, 2021, of the COVID-19 dataset. After estimating the consolidation distribution π_Δ_(*z*) relative to the date of reporting Δ (Figure 1A), we compute the corresponding consolidation lags *T*_Δ, *F*_ for different levels of completeness *F*, the nowcasted epidemic curve (Figure 1B), and the estimates of the reproduction numbers *R*_Δ_(*t*), ${\hat{R}}_{\Delta }\left(t\right)$, R^*^(*t*) (Figure 1C). This example shows that the net reproduction number significantly underestimated the reference value when the completeness value was below 90%. Thus, in this example the most recent reliable estimate obtainable on Δ = April 1, 2021 without nowcasting is relative to March 16, 2021, corresponding to a consolidation lag of *T*_Δ, 90_= 16 days before the date of reporting, whereas the nowcasted estimate would be reliable even for the day before (March 31, 2021). In the rest of the manuscript, this qualitative assessment is done quantitatively and for all reporting dates in the study period, with the aim of assessing the added value of applying nowcasting for situational awareness.

### Performance metrics

For each value of completeness *F* and each considered reporting date *D*, we calculated: the absolute error of the net and nowcasted estimates of the reproduction number against the corresponding reference value; the proportion of all reporting dates in which each estimate underestimated the reference value; and the proportion of all reporting dates for which either estimate was closer than the other to the reference value.

Additionally, we defined an “epidemic period” as a sustained period of time during which the reproduction number remains above the epidemic threshold of 1. Specifically, we defined the start of an epidemic period as the first of at least three consecutive reporting days where the estimate of the reproduction number was above 1; and the end of an epidemic period as the day before the first of at least seven consecutive reporting days where the estimate of the reproduction number was below 1. We compared the lags with which the net and nowcasted reproduction numbers were able to identify epidemic periods of duration longer than 15 days, as defined by the reference reproduction number.

## Results

**Figure 2:**
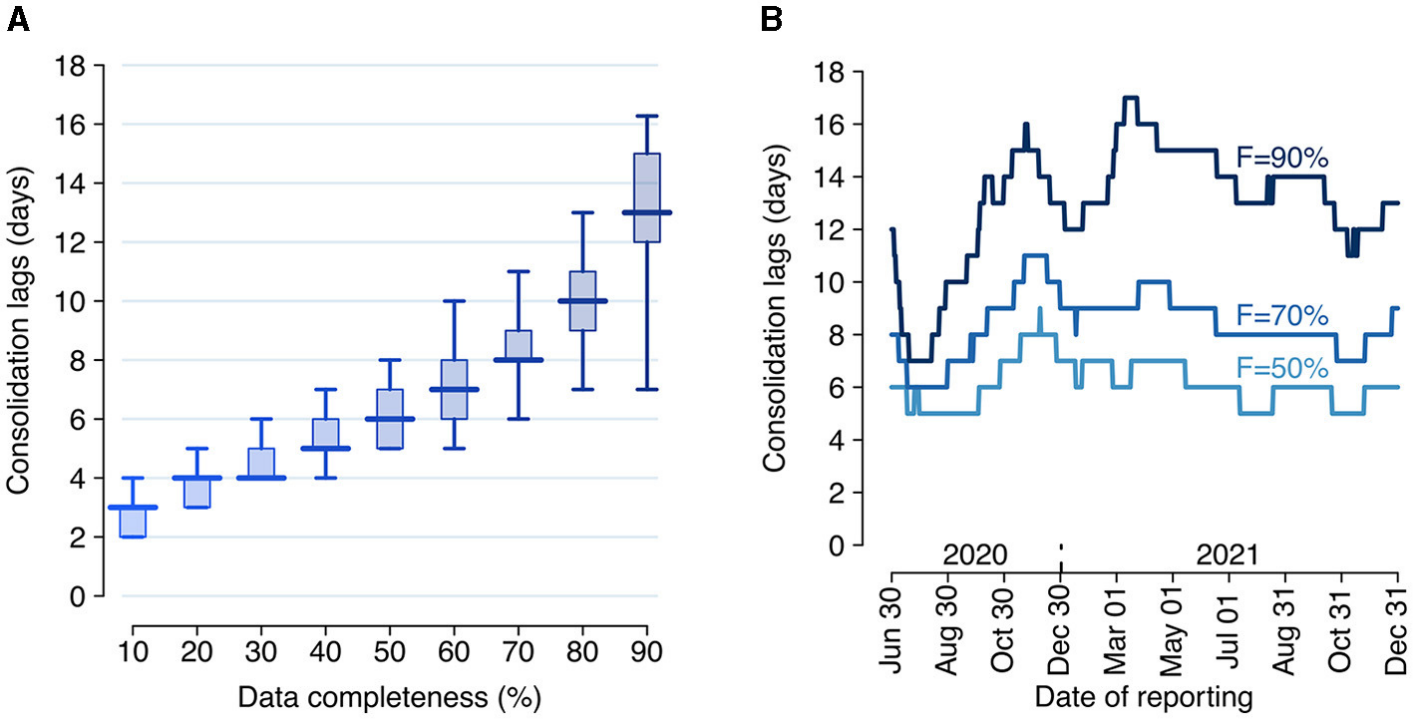
Consolidation lags for the Italian COVID-19 integrated surveillance system. **(A)** Distribution of the consolidation lag across the estimation period (June 29, 2020–December 31, 2021) for different values of completeness. Boxplots show the median (horizontal line), interquartile range (rectangle) and 95% quantiles (whiskers) over the *n* = 551 reporting dates. **(B)** Consolidation lags at different reporting dates as estimated for three selected values of completeness *F*.

During the study period, the Italian COVID-19 integrated surveillance system took about 6 days (median over the study period; 95% quantile: 5–8 days) from symptom onset to record at least 50% of all cases, and about 13 days (95% quantile: 7–17 days) to record at least 90% (Figure 2).

**Figure 3:**
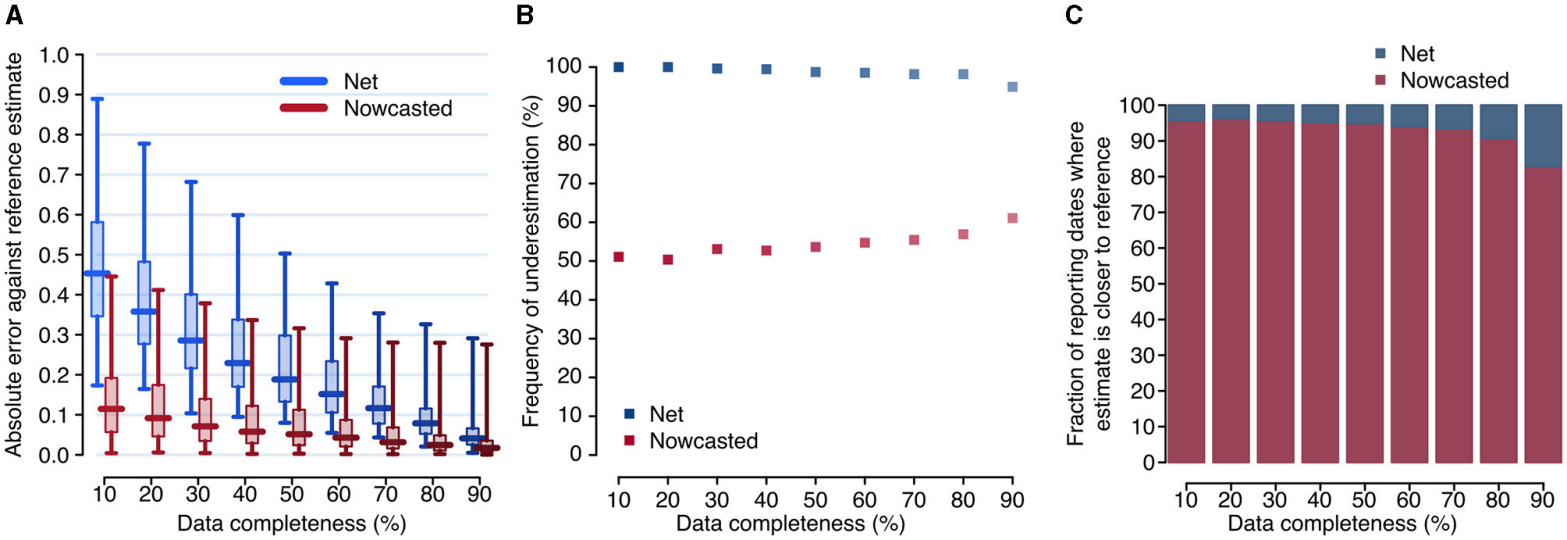
Accuracy of the net and nowcasted reproduction numbers. **(A)** Distributions of the absolute error between the reference reproduction number and the net and nowcasted reproduction numbers, computed at different reporting dates (daily between June 29, 2020, and December 31, 2021), and evaluated at the date corresponding to a specified level of completeness. Boxplots show the median (horizontal line), interquartile range (rectangle) and 95% quantiles (whiskers). **(B)** Fraction of reporting dates for which the net and nowcasted estimates of the reproduction number underestimate the reference value. **(C)** Fraction of reporting dates for which either estimate is the closest one to the reference value, for different values of completeness.

Overall, the net reproduction number was a good approximation of the reference reproduction number R^*^ when evaluated at the date of 90% completeness, with a median absolute error for *R*_90_ over the entire study period of 0.042 (interquartile range, IQR: 0.025–0.066, Figure 3). The corresponding nowcasted estimate ${\hat{R}}_{90}$ had a median absolute error of 0.017 (IQR: 0.007–0.036, significantly smaller than the error for *R*_90_: *p*-value of paired *t*-test between the errors of the net and nowcasted estimates ≪ 0.001).

When accepting lower thresholds for data completeness, the accuracy of the net reproduction number degraded rapidly, but not the one for nowcasted estimates. For example, with a completeness of 70% (corresponding to a median lag of 8 days), the median error was 0.116 (IQR: 0.078–0.171) for *R*_70_ but 0.032 (IQR: 0.016–0.068) for ${\hat{R}}_{70}$, i.e., still significantly smaller than the error for *R*_90_ (paired *t*-test *p*-value ≪ 0.001). The median error for ${\hat{R}}_{10}$ (0.115; IQR: 0.057–0.191), corresponding to a median lag of 3 days, was comparable to the median error for *R*_70_ (paired *t*-test *p*-value: 0.42). The net estimate systematically underestimated the reference value, while the nowcasted did so in about half of the reporting dates; the latter result was roughly independent on the considered completeness (Figure 3B). Furthermore, the nowcasted estimates were closer to the reference value, compared to net estimates obtained with the same completeness. This occurred for more than 90% of reporting dates when completeness values of 80% or lower were considered (Figure 3C).

**Figure 4:**
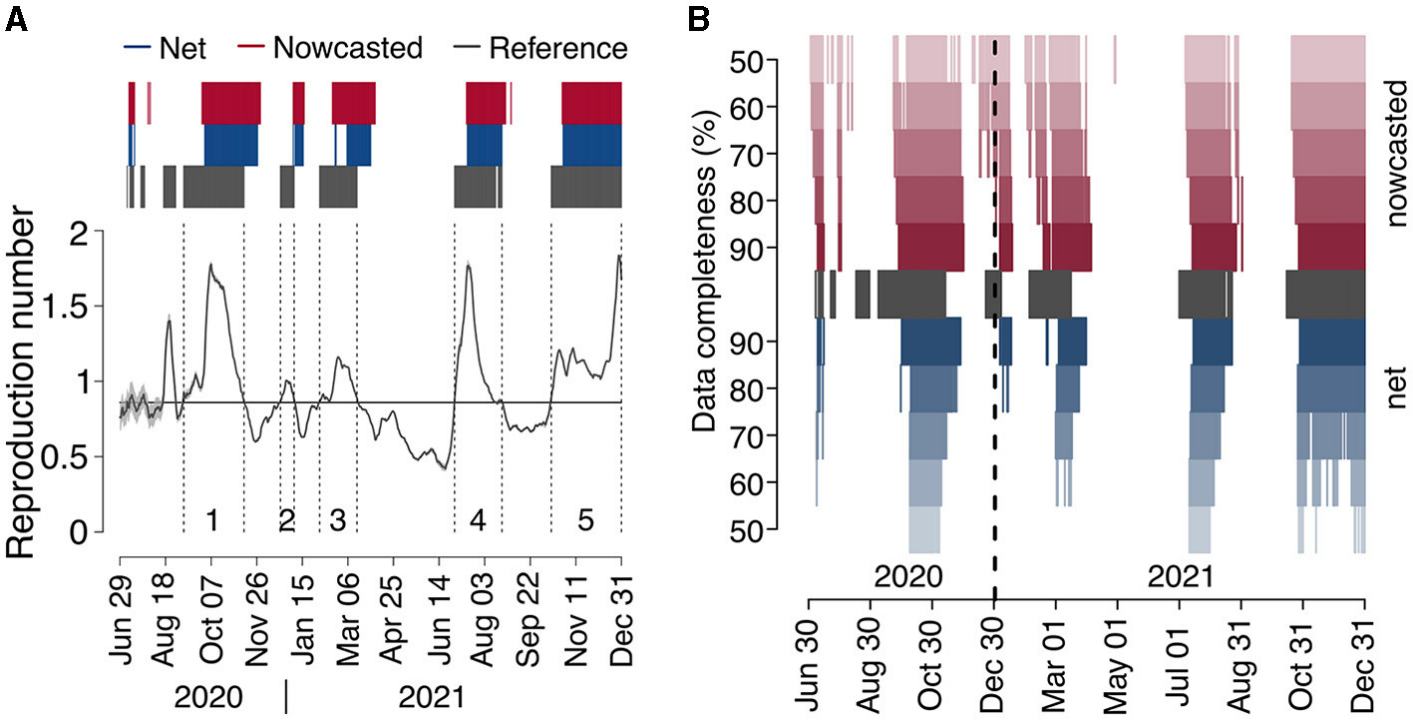
COVID-19 epidemic periods in Italy (2020–2021). **(A)** Reference reproduction number by date of symptom onset, as computed from consolidated data reported on April 5, 2023 (solid black line). The gray shaded area around the reference reproduction number (visible only in the period May–August 2020, due to the low number of cases contributing to R estimates) represents the 95% CI in its estimate. Dark gray bars above the graph highlight the days where the reference reproduction number was above 1, and dark gray vertical dashed lines delimit epidemic periods (see Table 1), labeled with a progressive number just above the *x*-axis. Blue and red bars identify the dates of reporting for which the net and nowcasted reproduction numbers, respectively, estimated at the nearest date afforded by a completeness of 90%, were above 1. **(B)** Dates where the reference reproduction number was above 1 (dark gray), and dates of reporting for which the net and nowcasted reproduction numbers, estimated at the nearest date afforded by a completeness between 50 and 90%, were above 1 (red and blue).

We identified five epidemic periods where the reference reproduction number was above the epidemic threshold for more than 15 days between June 29, 2020, and December 31, 2021 (Figure 4 and Table 1). The net estimate at 90% completeness detected the epidemic periods with a lag that ranged between 13 and 30 days (Table 1). The net estimate at 70% completeness reduced by 2–3 days the detection lag for epidemic periods 4–5 but increased the lag by 8 days for period 1 and missed the detection of period 2. The net estimate at 50% completeness missed the detection of three periods out of 5. The nowcasted estimate allowed anticipating the detection by 1–15 days (lags range: 12–20 days) at 90% completeness, and by 6–23 days (lags range: 7–17 days) at 70% completeness, compared to the net estimate at 90% completeness. In the case of 50% completeness, the nowcasted estimate performed worse than the net estimate at 90% completeness during period 1 (lag of 27 days vs. 23) but reduced the detection lag by 8 days for periods 4 and 5; for periods 2 and 3, the estimate provided an early warning 6 and 4 days compared to the actual start of the periods; however, it also falsely flagged an additional epidemic period between July 2 and August 13, 2020.

**Table 1:** Characteristics of COVID-19 epidemic periods in Italy (2020–2021) and detection lags due to consolidation of epidemic curves.

**Epidemic period**	**1**	**2**	**3**	**4**	**5**
Date of start	Sep 07, 2020	Dec 22, 2020	Feb 03, 2021	Jul 01, 2021	Oct 15, 2021
Date of end	Nov 12, 2020	Jan 06, 2021	Mar 16, 2021	Aug 22, 2021	After Dec 31, 2021
Duration (days)	67	16	42	53	>78
**Date of detection**					
Net (90% completeness)	Sep 30, 2020	Jan 07, 2021	Mar 05, 2021	Jul 15, 2021	Oct 28, 2021
Net (70% completeness)	Oct 8, 2020	–	Mar 01, 2021	Jul 12, 2021	Oct 26, 2021
Net (50% completeness)	Oct 10, 2020	–	–	Jul 11, 2021	–
Nowcasted (90% completeness)	Sep 27, 2020	Jan 05, 2021	Feb 17, 2021	Jul 14, 2021	Oct 27, 2021
Nowcasted (70% completeness)	Sep 24, 2020	Dec 30, 2020	Feb 10, 2021	Jul 09, 2021	Oct 22, 2021
Nowcasted (50% completeness)	Oct 4, 2020	Dec 16, 2020	Jan 30, 2021	Jul 07, 2021	Oct 20, 2021
**Detection lag (days)**					
Net (90% completeness)	23	16	30	14	13
Net (70% completeness)	31	–	26	11	11
Net (50% completeness)	33	–	–	10	–
Nowcasted (90% completeness)	20	14	14	13	12
Nowcasted (70% completeness)	17	8	7	8	7
Nowcasted (50% completeness)^*^	27	−6	−4	6	5

The dates of start and end of epidemic periods refer to dates in which the reference reproduction number (computed from consolidated data reported on April 5, 2023) was above 1 (see methods for the algorithmic definition). The dates of detection correspond to the first date of analysis for which the reproduction number, estimated at the date of the given completeness, was above 1.

^*^The nowcasted estimate at 50% completeness falsely flagged an additional epidemic period (July 2–August 13, 2020).

## Discussion

We performed a quantitative assessment of the use of nowcasting to improve situational awareness during an epidemic outbreak, based on extensive data from over 18 months of the COVID-19 epidemics in Italy. Nowcasting systematically outperformed the non-adjusted estimation of reproduction numbers, improving both the accuracy and timeliness of the estimates. In particular, the adopted nowcasting algorithm was able to more than halve the mean absolute error compared to the net estimate (i.e., from non-adjusted epidemic curves) when evaluated at the same median lag of 13 days before the date of analysis. Nowcasting maintained a better accuracy even for estimates relative to a median of only 8 days before the date of analysis. More importantly, the nowcasted estimates markedly reduced by between 6 and 23 days the lag in detecting the beginning of epochs of sustained epidemic circulation, a notoriously difficult task for forecasting approaches (30).

In Italy, official estimates of the COVID-19 reproduction number were based on net estimates with reference to 14 days before the date of reporting, which we found to approximately correspond to a data completeness of 90%. The analysis proposed here retrospectively validates the choice of a 14-days lag, given the remarkable worsening of accuracy that would be obtained with shorter lags. Official estimates were made available to the public through a weekly bulletin and used for decisions on non-pharmaceutical interventions (31). Nowcasted estimates of the reproduction number were additionally used by national and regional health authorities throughout the course of the emergency to gather further insights during weekly situation assessments, thus improving situational awareness. The analysis proposed here represents a retrospective validation of this additional estimate.

There are a few limitations that need to be considered when interpreting our results. First, the estimates of the reproduction numbers were obtained by assuming a fixed distribution of the generation time throughout the study period, corresponding to the one estimated for SARS-CoV-2 ancestral lineages. This choice was taken for simplicity and based on estimates for the Alpha (32), Delta (32) and Omicron variants (33) suggesting limited changes in the distribution of the SARS-CoV-2 generation time in Italy in the study period. Second, our main analysis compared different reproduction numbers only in terms of their mean estimate, without considering their variability. However, results remained robust when considering alternative error functions that considered the variability in estimates (see Supplementary material). For what concerns the definition of epidemic periods, we used a heuristic definition to compare the potential in early warning of the net and nowcasted estimates at different levels of completeness. This definition does not distinguish situations of moderate (R slightly above 1) vs. catastrophic (R much above 1) epidemic growth and therefore does not necessarily correspond to the need for public health decision makers to take urgent action. Still, the identified epidemic periods corresponded to the main periods of expansion of the COVID-19 epidemics in Italy during the study period (Figure 4A), including the second wave in the fall of 2020 (period 1), the short resurgence during Christmas holidays of 2020 (period 2), the wave related to the expansion of the Alpha variant in spring 2021 (period 3), the increase of cases in summer 2021, partially related to the celebrations for the Italian victory in Euro2020 soccer championship (34) (period 4), and the wave due to Delta in the fall of 2021, replaced by Omicron in the last week of the year (35) (period 5). Although daily updates of epidemic curves for SARS-CoV-2 were available until April 15, 2022 (after which updating over the weekends has been suspended), we decided to stop the benchmarking exercise on December 31, 2021, given the lower severity of the pandemic in 2022 (36–38), the broad diffusion of self-tests to be performed at home, and the progressive shift toward hospital surveillance. Finally, we were not able to assess the method on the first wave of the COVID-19 pandemic, due to the lack of harmonization in data collection across different regional health systems (especially in the earliest weeks after the first viral detection) and to the rapid temporal variations in ascertainment rates, organizational set-up in epidemiological investigations and data collection.

## Conclusions

The overall purpose of this study was to demonstrate and quantitatively assess the usefulness of a simple non-parametric nowcasting method from the perspective of situational awareness in real-time during epidemic outbreaks. Nowcasting can empower better informed public health responses through improved accuracy and timeliness of the estimates of the reproduction number and an earlier identification of periods of sustained epidemic growth. We suggest that nowcasting should become standard practice in surveillance activities, especially in situations of public health emergencies. Several methods have been proposed for nowcasting and some have been made publicly available as packages for statistical software (39–42). A systematic comparison of requirements, advantages and disadvantages, and performances against standardized might support epidemiological data analysts in choosing the most appropriate nowcasting tool for different situations. Although an assessment of the reliability of nowcasting in the early phase of the COVID-19 pandemic was not possible in our study, we argue that pandemic preparedness toward harmonized data collection can minimize the time until the condition is met (i.e., a stable surveillance system providing regular updates of observed epidemic curves) for a reliable application of nowcasting.

## Data Availability

Publicly available datasets were analyzed in this study. This data can be found at: https://www.epicentro.iss.it/coronavirus/sars-cov-2-sorveglianza-dati.
