# Supplementary material for: Increasing situational awareness through nowcasting of the reproduction number
Source: Front Public Health. 2024 Aug 21;12:1430920. doi: 10.3389/fpubh.2024.1430920 (PMC11371679; doi:10.3389/fpubh.2024.1430920)
Supplement: Supplementary file 1 [file Data_Sheet_1.pdf]

Supplementary materials

Increasing situational awareness through nowcasting of the reproduction number

Andrea Bizzotto [a,b,*], Giorgio Guzzetta [a,*,&], Valentina Marziano [a], Martina del Manso [c], Alberto Mateo Urdiales [c], Daniele Petrone [c], Andrea Cannone [c], Chiara Sacco [c], Piero Poletti [a], Mattia Manica [a], Agnese Zardini [a], Filippo Trentini [d,e], Massimo Fabiani [c], Antonino Bella [c], Flavia Riccardo [c], Patrizio Pezzotti [c,#], Marco Ajelli [f,#], Stefano Merler [a,#]

a Center for Health Emergencies, Bruno Kessler Foundation, Trento, Italy
b University of Trento, Italy
c Department of Infectious Diseases, Istituto Superiore di Sanità, Rome, Italy
d Covid Crisis Lab, Bocconi University, Milan, Italy
e Department of Social and Political Sciences, Bocconi University, Milan, Italy
f Laboratory for Computational Epidemiology and Public Health, Department of Epidemiology and Biostatistics, Indiana University School of Public Health, Bloomington, Indiana, United States

* Joint first authors
# Joint senior authors

1. A simple method for nowcasting epidemic curves 2
1.1 Definitions 2
1.2 Estimation of consolidation distributions and lags 2
1.3 Considerations on the choice of θ and N 3
1.4 A practical example 3
2. Integrated SARS-CoV-2 surveillance system 4
3. Estimation of the time-varying reproduction number 5
4. Retrospective assessment on the choice of θ 6
5. Sensitivity analysis on θ and N 7
6. Further results with alternative error functions 8
7. Alternative definition of the data consolidation lag 10
Supplementary references 12

1. A simple method for nowcasting epidemic curves

Here we provide the details of the nowcasting method used in this paper, which was developed within surveillance activities in the early months of the COVID-19 epidemic in Italy and is based on the same principles of the seminal paper by Lawless [S1].

1.1 Definitions

Given a generic infectious disease, we define D as a generic date of update of the epidemic curve by the epidemiological surveillance system. Therefore, D implicitly identifies different versions of the surveillance database. We define the "observed epidemic curve" $C_D(t)$ as the number of symptomatic cases C with symptom onset at date t reported by the surveillance system at a date of reporting D. We define the "consolidated epidemic curve" $C^*(t)$ as the epidemic curve that is observed at the end of the outbreak, when all cases have been inserted in the database. For dates of symptom onset sufficiently distant in the past from D, we can assume that the observed epidemic curve is consolidated, i.e., that there is a time interval θ such that for $t \leq D - \theta$, $C_D(t)$ equals $C^*(t)$. We thus define for each reporting date D a "consolidation distribution", π_D, representing the proportion of cases recorded in the consolidated curve C^* and with symptom onset dates between $D - \theta$ and D that were already reported at date D:

$$\pi_D(z) = \frac{C_D(D-z)}{C^*(D-z)}, \ (0 \leq z < \theta).$$

The consolidation distribution π_D can be interpreted also as the proportion of cases having symptom onset at date $t = D - z$ that are reported in the surveillance database within z days after symptom onset (i.e., at date D). By definition, π_D cannot be known exactly at the date of reporting D; however, if we can obtain an approximation $\hat{\pi}_D$ using data available until D, an approximation $\hat{C}_D(t)$ of $C^*(t)$ can be inferred at date D:

$C^*(t = D - z) \cong \hat{C}_D(t = D - z) = \frac{1}{\hat{\pi}_D(z)} C_D(t = D - z)$.We refer to $\hat{C}_D(t)$ as the "nowcasted epidemic curve" estimated at time D.

From the approximated $\hat{\pi}_D$, we can additionally define a "consolidation lag" $T_{D,F}$, representing the minimum number of days that need to elapse before the number of cases of a given symptom onset date exceeds a given fraction F of all cases that will be recorded at the end of the outbreak:

$$T_{D,F} = \min\{z: \hat{\pi}_D(z) \geq F\}$$

The consolidation lag gives an indication on until which symptom onset date the number of cases observed at time D can be considered sufficiently complete; for example, we can consider the epidemic curve observed at time D to be at least 90% complete until symptom onset date $t \leq D - T_{D,90}$.

1.2 Estimation of consolidation distributions and lags

One way to estimate $\hat{\pi}_D(z)$ is to rely on consolidation distributions relative to symptom onset dates $t \leq D - \theta$ that are consolidated at the date of reporting D. These consolidation distributions can be obtained by comparing the number of cases for a given symptom onset date across successive reporting updates. For example, for $t = D - \theta$ we will have:

$$p_{D,0}(z) = \frac{C_{D-\theta+z}(D-\theta)}{C^*(D-\theta)} = \frac{C_{D-\theta+z}(D-\theta)}{C_D(D-\theta)}$$

since we have assumed that $C_D(t = D - \theta) = C^*(t = D - \theta)$. Equivalently, for any symptom onset date $t = D - \theta - i, i \geq 0$:

$$p_{D,i}(z) = \frac{C_{D-\theta-i+z}(D-\theta-i)}{C_{D-i}(D-\theta-i)}$$

We propose to approximate the unknown consolidation distribution $\pi_D(z)$ with the average over consolidation distributions $p_{D,i}$ relative to the N symptom onset dates closest to D that are consolidated at D:

$$\hat{\pi}_D(z) = \frac{1}{N} \sum_{i=0}^{N-1} p_{D,i}(z)$$

1.3 Considerations on the choice of θ and N

The appropriate choice of the values for θ and N may not be obvious. The correct value of θ is itself dependent on the same consolidation lags that we want to estimate, and represents an abstraction, as there is no theoretical time limit with which an epidemic curve may be retrospectively updated. An educated guess of an appropriate value can be given by observing the consolidation lags related to different symptom onset dates in the early phases of the epidemic under study. For what concerns the value of N, it should be sufficiently high to stabilize the variability of observable consolidation distributions; however, the higher is N, the older is the information included in the estimation of consolidation distributions. Note also that, with the present method, the first nowcasted estimates can be produced at least $\theta + N - 1$ days after the first date of reporting available. For the application to COVID-19 data, we chose θ = 30 days and N = 30, and subjected this choice to a retrospective assessment in Section 4 and to a sensitivity analysis in section 5 of the present document.

1.4 A practical example

Figure S1 reports a practical example of the proposed method to approximate the consolidation distribution for a specific reporting date $\Delta =$ April 1, 2021, of the COVID-19 dataset. The closest date of symptom onset for which the number of cases can be assumed to be consolidated (i.e., numbers for this date will not change in reported epidemic curves successive to April 1, 2021) is $\Delta - \theta$ = March 2, 2021, given our choice of θ=30 days. The estimate of $p_{\Delta,0}$ will be given by the number of cases with symptom onset on March 2 reported in epidemic curves produced between March 2 and April 1 and normalized by the consolidated number reported on April 1 (Figure S1a). The estimate of $p_{\Delta,N-1}$, for N=30, will be given by the number of cases with symptom onset on $\Delta - \theta - (N - 1) =$ February 1, reported between February 1 and March 3, normalized by the number reported on March 3. After averaging the N observed consolidation distributions, we obtain the consolidation distribution $\hat{\pi}_\Delta(z)$ relative to the date of reporting Δ (Figure S1b). Figure 1 in the main text shows an example of using the consolidation distribution to compute the nowcasted epidemic curve and reproduction numbers for the same date of reporting.

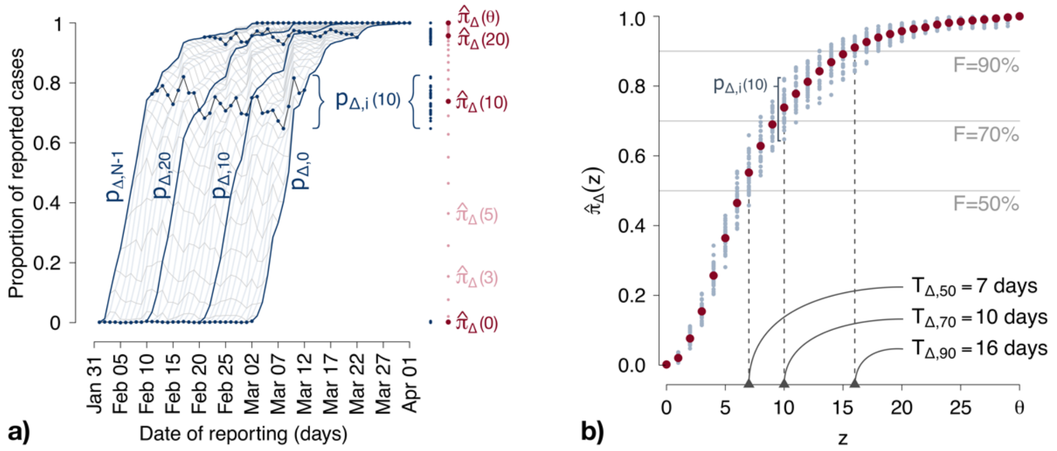

Figure S1. Example of application of the proposed nowcasting technique using data from the Italian COVID-19 integrated surveillance system for a selected reporting date ($\Delta =$ April 1, 2021). a) Approximation of the consolidation distribution. Light blue lines represent consolidation distributions for symptom onset dates between February 1 ($p_{\Delta,N-1}$) and March 2 ($p_{\Delta,0}$), 2021; $p_{\Delta,0}, p_{\Delta,10}, p_{\Delta,20}$ and $p_{\Delta,N-1}$ are reported in thicker and darker lines to highlight the variability across consolidation distributions. Dotted blue lines connect the values of different consolidation distributions for the same number z of days elapsed since the corresponding symptom onset, $p_{\Delta,i}(z)$, for z=0, 10, 20 and θ (gray lines show $p_{\Delta,i}(z)$ for the other values of z). The $p_{\Delta,i}(z)$ distributions for the selected values of z are reported on the right-hand side of the panel as blue dots, and their mean, corresponding to the estimated value of $\hat{\pi}_\Delta(z)$, is reported further to the right as a dark red dot. Pink dots represent the mean of $p_{\Delta,i}(z)$ distributions, corresponding to the estimated value of $\hat{\pi}_\Delta(z)$, for the remaining values of z. b) Estimated consolidation distribution and corresponding consolidation lags. Light blue dots represent the distributions of $p_{\Delta,i}(z)$ for all values of z; dark red dots represent the estimated consolidation distribution at Δ, $\hat{\pi}_\Delta(z)$. Horizontal lines define selected completeness thresholds (50%, 70% and 90%) and corresponding vertical lines define the corresponding consolidation lags.

2. Integrated SARS-CoV-2 surveillance system

Since February 27, 2020, the Istituto Superiore di Sanità has coordinated a national case-based surveillance system reporting on all human cases with laboratory-confirmed SARS-CoV-2 infections [S2] defined as per the concurrent European Case Definition [S3]. Until January 2021, all cases were confirmed by RT-PCR; after that date, confirmation with rapid antigen tests was also accepted [S4]. The system was mandated by national law, regulated by dedicated technical documents that described data and data quality requirements and provided weekly data quality verification reports and ad hoc verification of incomplete/inconsistent items to each of the 21 Italian Regions and Autonomous Provinces [S5].
The system was based on an online dedicated and secure platform initially compiled manually by regional public health officers. As the epidemic progressed, with high case-loads, standardization for the upload of regional datasets was defined and implemented. The system allowed the collection of demographic data, geographic location data, date of symptom onset, date of diagnoses, date of hospitalization as well data on clinical severity and outcome. The definition of a case as symptomatic and of its date of symptom onset included any SARS-CoV-2-related symptoms, independently of its severity, as defined by clinical evaluation of the case. The definition of a case as imported was based on exposure outside the Italian territory ascertained through epidemiological investigations.
Results of the surveillance system were summarized in a daily dashboard, epidemiological bulletins and used as one of the sources of a mixed method risk assessment protocol that supported pandemic response in Italy [S6].

3. Estimation of the time-varying reproduction number

The estimation of the time-varying reproduction number was performed by applying a standard method [S7–9] which requires as input the number of autochthonous (locally transmitted) cases by date of symptom onset $\mathrm{A(t)}$, the number of imported cases (infections acquired outside the geographical setting of interest) $\mathrm{I(t)}$, and an estimate of the distribution of the generation time $\varphi(\mathrm{t})$ for the infection under study. The posterior distribution of $\tilde{\mathrm{R}}(\mathrm{t})$ was obtained by applying Markov Chain Monte Carlo with Metropolis-Hasting sampling ($\mathrm{n} = 50{,}000$ iterations) to the likelihood function defined below:

$$\mathcal{L} = \prod_{t=1}^{D} P\left(A(t);\ \tilde{R}(t) \sum_{s=1}^{t} \varphi(s) C(t-s)\right)$$

where:

- $\mathrm{P(k;\ \lambda)}$ is the probability mass function of a Poisson distribution (i.e., the probability of observing k events if these events occur with rate λ).
- $\mathrm{C(t) = A(t) + I(t)}$ is the total epidemic curve (total number of cases with symptom onset at time t).
- $\varphi(\mathrm{s})$ is the integral of the probability density function of the generation time evaluated between day s-1 and s.
- $\tilde{\mathrm{R}}(\mathrm{t})$ is the daily reproduction number at time t.

The posterior distribution of the time-varying reproduction number $\mathrm{R(t)}$ was computed by applying a weekly moving average to the posterior distribution of $\tilde{\mathrm{R}}(\mathrm{t})$.
The same procedure reported above was applied to the consolidated epidemic curves $\mathrm{C^*(t)}$ in order to estimate the reference reproduction number $\mathrm{R^*(t)}$, and, at each reporting date D, to the net and nowcasted epidemic curves ($\mathrm{C_D(t)}$ and $\hat{\mathrm{C}}_\mathrm{D}(\mathrm{t})$ respectively), in order to estimate the net ($\mathrm{R_D(t)}$) and nowcasted ($\hat{\mathrm{R}}_\mathrm{D}(\mathrm{t})$) reproduction numbers over time.
For application to the Italian COVID-19 data, nowcasting was applied only to the autochthonous component of the epidemic curve, based on the observation that imported cases represented in most cases a negligible fraction of the total (median 0.4%, IQR 0.20-1.88% across symptom onset dates between January 28, 2020, and December 31, 2021). For the distribution of the generation time $\varphi(\mathrm{s})$, we used the distribution of the serial interval estimated in Italy for ancestral lineages [S10], given by a gamma function with shape 1.87 and rate 0.28, for a mean of 6.68 days. Further estimates of the distribution of the generation time in Italy for successively emerged variants showed minimal changes with respect to these values [S10, S11].

4. Retrospective assessment on the choice of θ

A critical assumption in the method for nowcasting epidemic curves is that, for $t \leq D - \theta$, the reported epidemic curve at day D, $C_D(t)$, approximates the consolidated epidemic curve $C^*(t)$. In order to assess the goodness of the chosen value for θ (equal to 30 days in the baseline analysis) when nowcasting COVID-19 epidemic curves in Italy, we computed the mean absolute percentage error $\eta(\theta)$ and the root mean squared error $\varepsilon(\theta)$ between the last values considered stable for the reported epidemic curve, given by $\tilde{C}(t;\theta) = C_{t+\theta}(t)$ at a certain value of θ, and the corresponding reference values $C^*(t)$:

$$\eta(\theta) = \frac{1}{n}\sum_{t=t_0}^{T} \frac{\left|\tilde{C}(t;\theta) - C^*(t)\right|}{C^*(t)}$$

$$\varepsilon(\theta) = \sqrt{\frac{1}{n}\sum_{t=t_0}^{T} \left|\tilde{C}(t;\theta) - C^*(t)\right|^2}$$

Where t_0 and T are the first and last symptom onset dates over which the error is computed and $n = T - t_0 + 1$ is the number of data points in $\tilde{C}(t)$. We evaluated $\eta(\theta)$ and $\varepsilon(\theta)$ for θ between 1 and 50, over the period comprised between t_0 = May 1, 2020 (the first reporting date that was available in our dataset) and T = November 11, 2021 (the last date for which $\tilde{C}$ could be computed when θ=50), resulting in n=560 data points. Figure S2 shows that both error metrics stabilize when θ is above 20 days, confirming that θ=30 days was an appropriate choice for Italian COVID-19 data.

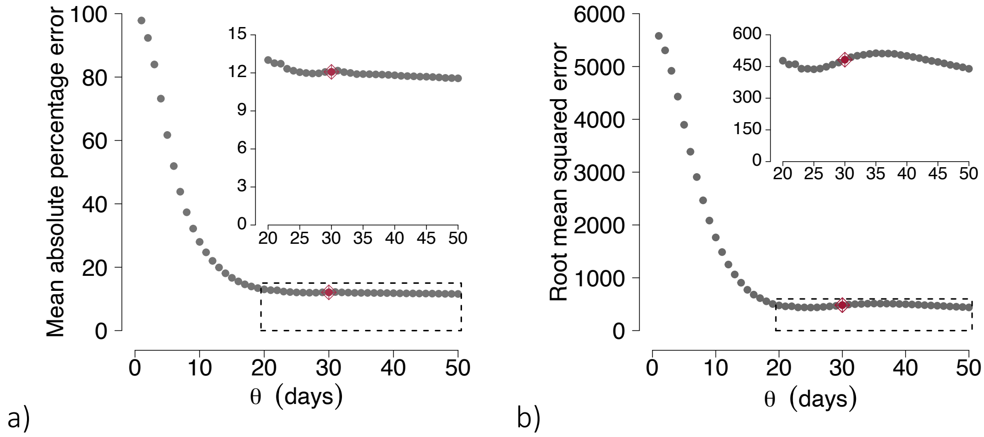

Figure S2. Error metrics for assessing the choice of $\boldsymbol{\theta}$. a) Mean absolute percentage error, $\eta(\theta)$; b) Root mean square error, $\varepsilon(\theta)$. The error value for the adopted value of θ=30 days is highlighted in red. To better appreciate differences in errors for values of θ>20 days, insets show the same graph with a zoom on the y-axis.

5. Sensitivity analysis on θ and N

The performance of the method proposed in this study may depend on the choice of its two parameters: the number of days after which a symptom onset date can be considered consolidated (θ) and the number of observed consolidation distributions used to nowcast the epidemic curves (N). In order to evaluate the sensitivity of model results with respect to the chosen values, we repeated the whole analysis for 6 additional combinations of N and θ, where both parameters can assume values of 20, 30 and 40. Since the first nowcasted estimates are available $\theta + \mathrm{N} - 1$ days after the date of the first dataset, we evaluated results for reporting dates that were common to all combinations of θ and N, i.e. between July 19, 2020 (first date of availability of nowcasted estimates for $\mathrm{N} = \theta = 40$) and December 31, 2021. Figures S3-S4 show minimal changes in the estimation of consolidation lags and in the performance of the method when choosing alternative values of the two parameters for selected data completeness levels (F = 50%, 70% and 90%).

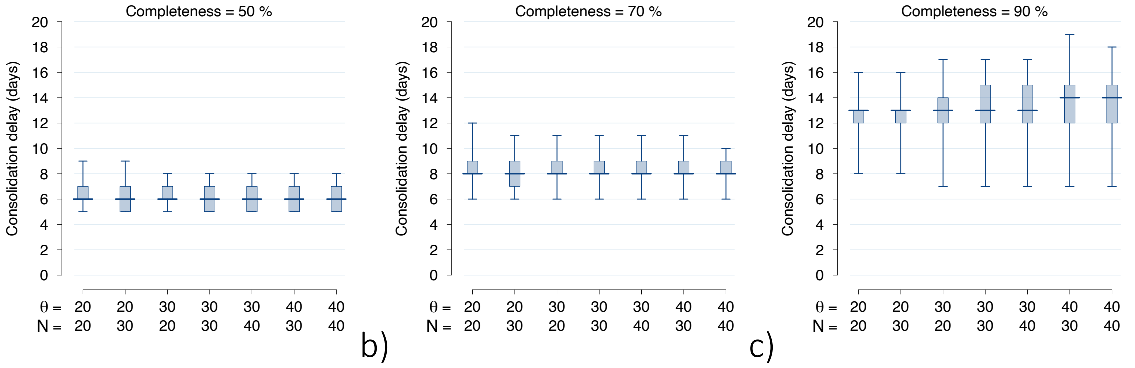

Figure S3. Distribution of consolidation lags for different combinations of the parameters **θ** and N. a) 50% completeness. B) 70% completeness. C) 90% completeness.

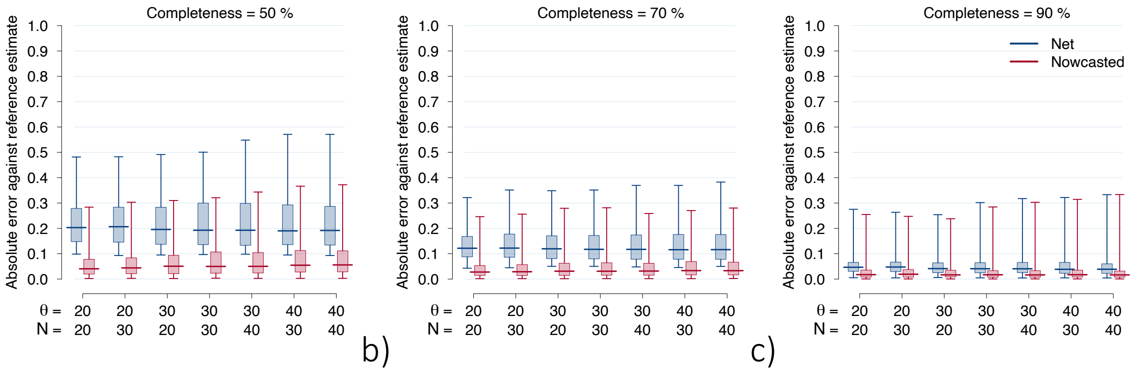

Figure S4. Distribution of the absolute errors between the reference reproduction number and the net and nowcasted reproduction numbers, respectively, for different combinations of parameters θ and N and for selected levels of completeness. a) 50% completeness. B) 70% completeness. C) 90% completeness.

6. Further results with alternative error functions

In this section, we report results on further performance metrics for the net and nowcasted reproduction numbers in the baseline analysis (θ=30 days, N=30).
Given the net reproduction numbers $R_F(D) = R(D - T_{D,F})$ estimated for a given data completeness F and at each reporting date D and consolidation lag $T_{D,F}$, we considered:
i) the bias error:

$$E_F^B(D) = R^*(D) - R_F(D),$$

ii) the percentage error:

$$E_F^P(D) = 100 \cdot \frac{|R_F(D) - R^*(D)|}{R^*(D)},$$

iii) the error with tolerance:

$$E_F^T(D) = \max\{R_F(D) - R_{97.5}^*(D);\ R_{2.5}^*(D) - R_F(D);\ 0\},$$

where $R_{2.5}^*$ and $R_{97.5}^*$ are respectively the lower and upper bounds of the 95%CI for the posterior distribution of R^*. The error with tolerance is defined in such a way to be zero when the average value of the net reproduction is within the 95% confidence interval of the reference estimate, and to be equal to the distance between the estimate and the closest boundary of the confidence interval otherwise.

The corresponding errors were computed for the nowcasted reproduction numbers by substituting R_F with $\widehat{R}_F$.
Figure S5 and Table S1 compare the overall performance of the net and nowcasted estimates for different values of data completeness. The bias error shows a significant downward bias (underestimation) for the net reproduction number and a minimal bias for the nowcasted reproduction number at all levels of data completeness (see also Figure 3b in the main text). The percentage error shows that the net estimate at 90% completeness and the nowcasted estimate at 70% completeness approximate the reference value with an accuracy higher than 95% in a majority of reporting dates. The results of the error with tolerance may be better appreciated in Figure S6 and Table S2, where we considered the fraction of times in which the net and nowcasted estimates are:

i) correct (when the mean estimate falls within the 95%CI of the reference);
ii) underestimated (when the mean estimate falls below the 95%CI of the reference);
iii) overestimated (when the mean estimate falls above the 95%CI of the reference).

Net estimates of the reproduction number are correct only for 9% of reporting dates at a data completeness level of 90%, against 26% for the corresponding nowcasted estimate. Even with a data completeness of 50%, the nowcasted estimates are correct in a higher proportion of reporting dates (14%) compared to the net estimate at 90%.

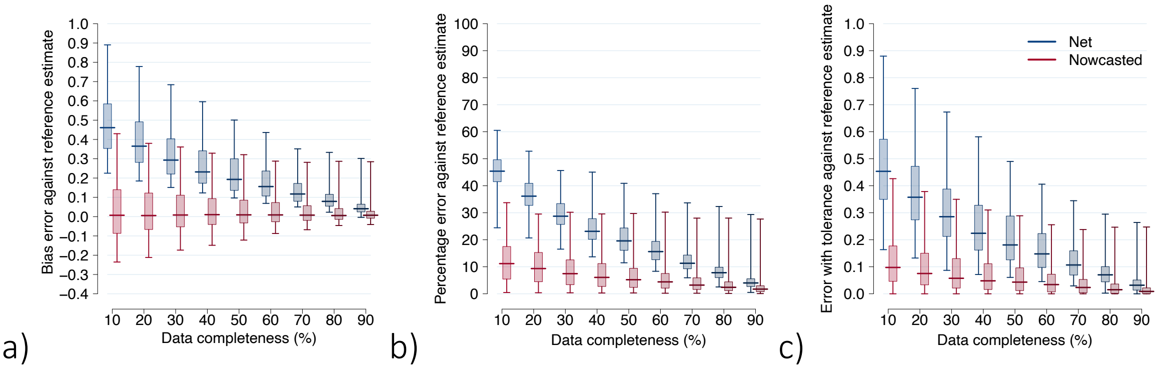

Figure S5. Comparison of performance between the net and nowcasted estimates using alternative error functions. a) Bias error; positive values represent underestimations of the reproduction number. b) Percentage error. c) Error with tolerance on the statistical variability of the reference estimate.

Estimate	**F** (%)	Absolute error (median and IQR)	Bias error (median and IQR)	Percentage error (median and IQR)	Error with tolerance (median and IQR)
Nowcasted	50	0.051 [0.023, 0.109]	0.008 [-0.035, 0.084]	5.3% [2.7, 9.8]	0.044 [0.012, 0.097]
	70	0.032 [0.016, 0.068]	0.006 [-0.021, 0.055]	3.4% [1.6, 6.2]	0.023 [0.003, 0.054]
	90	0.017 [0.007, 0.036]	0.007 [-0.009, 0.028]	1.8% [0.8, 3.1]	0.009 [0, 0.022]
Net	70	0.116 [0.078, 0.171]	0.116 [0.078, 0.171]	11.2% [9.1,14.3]	0.105 [0.067, 0.158]
	90	0.042 [0.025, 0.066]	0.039 [0.023, 0.064]	4.0% [2.7, 5.6]	0.029 [0.012, 0.049]

Table S1. Comparison of performance metrics for the net and nowcasted estimates for selected levels of data completeness.

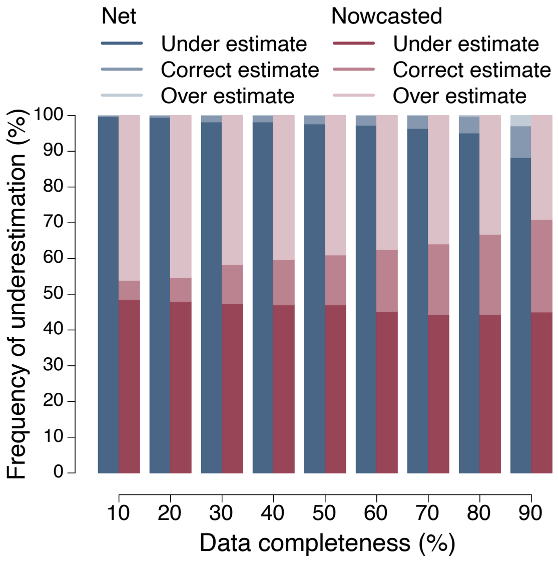

Figure S6. Performance of the net and nowcasted reproduction numbers when considering the stochastic variability of the reference estimate, for different data completeness levels.

Method	Data completeness (%)	Under-estimate %	Over-estimate %	Correct estimate %
Nowcasted	50	47	39	14
	70	44	36	20
	90	45	29	26
Net	90	88	3	9

Table S2. Performance of the net and nowcasted reproduction numbers when considering the stochastic variability of the reference estimate, for selected data completeness levels.

7. Alternative definition of the data consolidation lag

In the main text, we considered explicitly the case in which consolidation distributions $\hat{\pi}_\Delta(z)$ are strictly monotonic with z (Figure 1 and S1). However, consolidation distributions in actual data may be non-monotonic when the process of consolidation due to reporting delays is counterbalanced by the retrospective removal of cases associated to a date of symptom onset. This may happen for a number of reasons, such as the re-evaluation of the date of symptom onset for a case after additional epidemiological investigations or after identification of data entry errors, the reclassification of cases as non-cases, or as asymptomatic cases, the identification of case duplications in the dataset, and so on. In these situations, the functions $\hat{\pi}_\Delta(z)$ may exceed the value of 1 at some z between 0 and θ. Figure S6 shows a comparison of the consolidation distribution for two reporting dates: July 15, 2020, and July 15, 2021. We evaluated the effect of an alternative definition of the data consolidation lag $T_{\Delta,F}$ where we considered, instead of the earliest z for which $\hat{\pi}_\Delta(z)$ reaches the desired completeness F, the latest z for which $\hat{\pi}_\Delta(z)$ is less than $1 - F$ away from its ideal value of 1:

$$T'_{\Delta,F} = \max_z\{|1 - \hat{\pi}_\Delta(z)| \leq 1 - F \ \wedge \ |1 - \hat{\pi}_\Delta(z-1)| > 1 - F\}.$$

An example of this second definition is shown in Figure S7 for July 15, 2020, and F=90%.

In Figure S8, differences in the distribution of the data consolidation lag when considering the alternative definition are shown for different values of F and limited to the 210 reporting dates with a non-monotonic consolidation distribution. For F<70%, the data consolidation lags are identical; for F=80% and 90% the alternative definition produces longer data consolidation lags, but with substantially similar medians. In particular, with a data completeness of 80%, the alternative definition yields the same median lag of 10 days but with a 95% confidence interval shifted upward (7-22 days versus 6-13 days in the baseline); with a data completeness of 90% the median lag grows from 12 days (95%CI 7-15) in the baseline to 13 days (95%CI 8-26) in the alternative definition. Table S3 shows that the alternative definition improves the performance of both the net and nowcasted estimates at 80% and 90% completeness, although at the cost of higher lags.

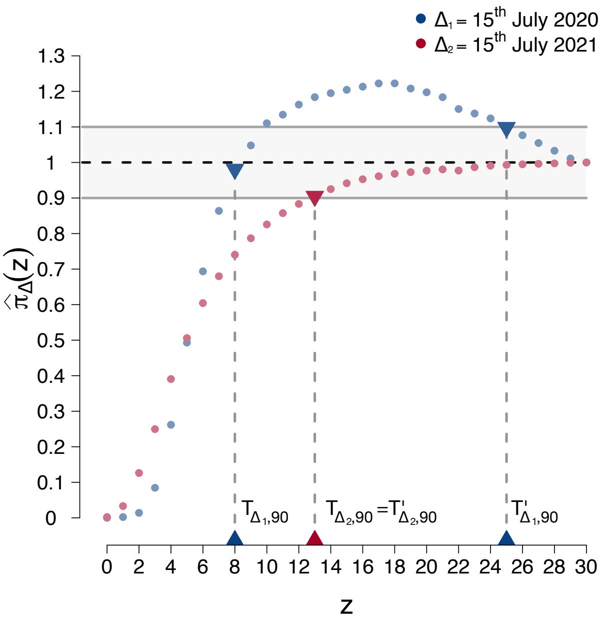

Figure S7. Example of alternative definitions for the data consolidation lag. Blue and red dots represent the consolidation distributions associated to reporting dates July 15, 2020, and July 15, 2021, respectively. Triangles on the x-axis represent the estimated data consolidation lags according to the two definitions. In the case of a monotonic curve (Δ_2 = July 15, 2021), the two definitions are equivalent, while when the curves are non-monotonic (Δ_1 = July 15, 2020), the two definitions may give different results.

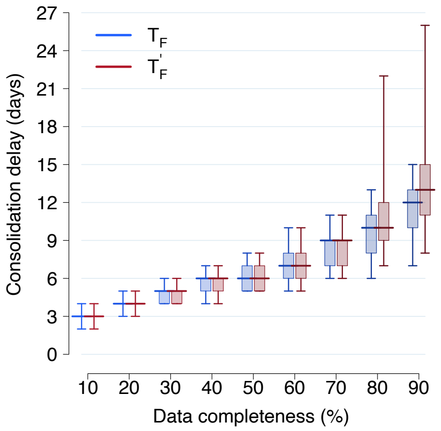

Figure S8. Impact of alternative definitions for the data consolidation lag. The figure shows the data consolidation lag distributions for different levels of data completeness, according to the baseline and alternative definition.

Method	Data completeness (%)	Definition of data consolidation lag	Absolute error (median and IQR)
Nowcasted	80	Baseline	0.048 [0.02, 0.106]
	80	Alternative	0.044 [0.019, 0.102]
	90	Baseline	0.041 [0.018, 0.091]
	90	Alternative	0.031 [0.016, 0.07]
Net	80	Baseline	0.083 [0.055, 0.168]
	80	Alternative	0.074 [0.041, 0.148]
	90	Baseline	0.054 [0.027, 0.099]
	90	Alternative	0.041 [0.018, 0.093]

Table S3. Absolute errors of the nowcasted and net reproduction numbers against the reference estimate, using alternative definitions of the data consolidation lag, for data completeness levels of 80% and 90%.

Supplementary references

S1. Lawless, J. f. Adjustments for reporting delays and the prediction of occurred but not reported events. *Canadian Journal of Statistics* **22**, 15–31 (1994).

S2. Riccardo, F. *et al.* Epidemiological characteristics of COVID-19 cases and estimates of the reproductive numbers 1 month into the epidemic, Italy, 28 January to 31 March 2020. *Euro Surveill* **25**, 2000790 (2020).

S3. Case definition for COVID-19. https://www.ecdc.europa.eu/en/infectious-disease-topics/z-disease-list/covid-19/facts/case-definition-covid-19 (2023).

S4. Italian Ministry of Health. Circolare n. 0705 "Aggiornamento della definizione di caso COVID-19 e strategie di testing" (Update on the case definition of COVID-19 and testing strategies), January 8, 2021.

S5. COVID-19, M. D. M., Xanthi Andrianou, Alberto Mateo Urdiales, Maria Fenicia Vescio, Maria Cristina Rota, Massimo Fabiani, Stefano Boros, Stefania Bellino, Paola Stefanelli, Alessandra Ciervo, Ornella Punzo, Antonietta Filia, Marco Tallon, Corrado Di Benedetto, Matteo Spuri, Patrizio Pezzotti, Flavia Riccardo, Antonella Bella, Gruppo Referenti regionali della Sorveglianza Integrata. La sorveglianza integrata Covid-19 in Italia: output e attività correlate. *www.epiprev.it* https://epiprev.it/5088.

S6. Riccardo, F. *et al.* COVID-19 response: effectiveness of weekly rapid risk assessments, Italy. *Bull World Health Organ* **100**, 161–167 (2022).

S7. Cori, A., Ferguson, N. M., Fraser, C. & Cauchemez, S. A New Framework and Software to Estimate Time-Varying Reproduction Numbers During Epidemics. *American Journal of Epidemiology* **178**, 1505–1512 (2013).

S8. Thompson, R. N. *et al.* Improved inference of time-varying reproduction numbers during infectious disease outbreaks. *Epidemics* **29**, 100356 (2019).

S9. Zhang, J. *et al.* Evolving epidemiology and transmission dynamics of coronavirus disease 2019 outside Hubei province, China: a descriptive and modelling study. *Lancet Infect Dis* **20**, 793–802 (2020).

S10. Cereda, D. *et al.* The early phase of the COVID-19 epidemic in Lombardy, Italy. *Epidemics* **37**, 100528 (2021).

S11. Manica, M. *et al.* Estimation of the incubation period and generation time of SARS-CoV-2 Alpha and Delta variants from contact tracing data. *Epidemiol Infect* **151**, e5 (2022).

S12. Manica, M. *et al.* Intrinsic generation time of the SARS-CoV-2 Omicron variant: An observational study of household transmission. *Lancet Reg Health Eur* **19**, 100446 (2022).
